# Editorial: Current Views of Hypothalamic Contributions to the Control of Motivated Behaviors

**DOI:** 10.3389/fnsys.2019.00032

**Published:** 2019-08-13

**Authors:** Joel D. Hahn, George Fink, Menno R. Kruk, B. Glenn Stanley

**Affiliations:** ^1^Department of Biological Sciences, University of Southern California, Los Angeles, CA, United States; ^2^Florey Institute of Neuroscience and Mental Health, The University of Melbourne, Melbourne, VIC, Australia; ^3^Leiden Academic Centre for Drug Research (LACDR), Leiden University, Leiden, Netherlands; ^4^Department of Molecular, Cell and Systems Biology, University of California, Riverside, Riverside, CA, United States

**Keywords:** hypothalamus, brain, motivated behavior, innate behavior, sensory - motor coordination

## What are motivated behaviors?

The goal of this Research Topic was to assemble a diverse collection of current views of the hypothalamus relating to its role in the control of motivated behaviors. This editorial highlights the included articles directly and also indirectly via two perspectives (from George Fink and Menno Kruk) that frame the topic in a historical context. However, before these, it is apt to reconsider briefly what is meant by the term “motivated behaviors.”

According to the Oxford English Dictionary, the noun “motivation” (from adjective “motive”) stems from the Latin *movēre*, meaning “to move^1^,” and the noun “behavior” (from the verb “behave”) stems from a combination of “be-” (as a prefix) and “have,” conveying “to have or bear oneself (in a specified way),” that is to conduct oneself intentionally^2^. Motivated behaviors may then be thought of literally as the expression of intentional (or purposeful) movements. This understanding is reflected in their common description of being oriented, directed, or driven by a goal.

**Figure 1:**
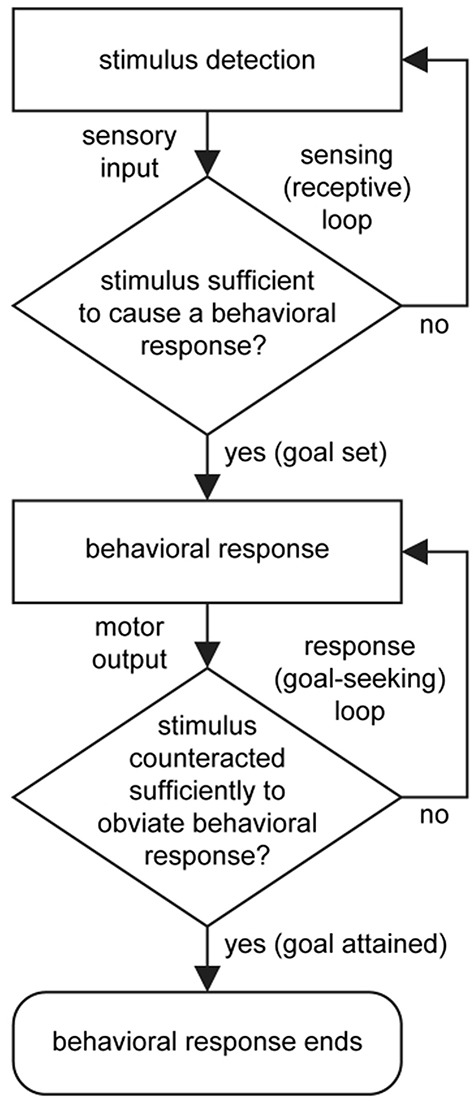
Basic flow diagram for the control of behavior. A stimulus is detected and transmitted (sensory input). If the stimulus is sufficient to cause a behavioral response, then a motor output will ensue. If the response sufficiently counteracts the originating stimulus then the behavioral response ends. Two stages of processing are looped: The first is a sensing (or receptive) loop that confers vigilance, and the second is a response (or goal-seeking) loop that enables error correction. Fundamentally, the setting, seeking, and attaining of a goal is a determined by interaction between the sensory and motor divisions of the nervous system. Note that “selection” of a behavioral response occurs when a stimulus reaches the response threshold, and that temporally, multiple sensing loops, responsive to different sensory stimuli, operate in parallel and concurrently with behavioral output.

From a neuroscientific standpoint, the terms goal-oriented, goal-directed, and goal-driven, all convey essentially the same basic idea that orientation, direction, or drive toward a goal (that which motivates) occurs when a change in the internal (body) or external environment that is detected by the sensory division of the nervous system achieves a level of input stimulation that is sufficient to activate a behavioral output response from the body via the motor division of the nervous system. A goal is attained when the behavioral response counteracts the originating stimulus to a level at which it no longer stimulates the behavioral response (Figure 1). Examples include the drive to regulate body temperature, fluid balance, and energy status in response to sensed changes in these, in order to maintain homeostasis (Watts and Swanson, 2002).

Through a process of natural selection, animals have evolved motivated behaviors that support the life goals of survival and reproduction, and the motivated behaviors that fundamentally support these goals include those for which the hypothalamus plays a central role: ingestive (eating and drinking), agonistic (defensive and aggressive), sexual, and allied to these the control of behavioral state (the level of intrinsic behavioral arousal) (Swanson, 1987; Simerly, 2015).

In seeking to understand motivated behaviors, it is noteworthy that the distinction between movement *per se* and purposeful movement that is considered motivated behavior, is neither obvious nor absolute. For example, reflexes such as the patellar stretch reflex (knee-jerk) are not typically thought of as motivated behaviors, but they do involve movement that is ostensibly purposeful (postural retention in the case of the patellar reflex). Nevertheless, behaviors can to some extent be classified according to the parts of the nervous system that are necessary and sufficient for their expression.

Voluntary (cognitive) control of behavior requires the cerebral cortex; whereas control of innate (instinctive) behaviors is classically associated with the hypothalamus. At the lowest hierarchical level are reflex behaviors, such as the patellar reflex that involves a monosynaptic reflex arc between sensory and motor neurons in the spinal cord. Classic lesion experiments have shown that innate behaviors can be performed to some extent without the cerebral cortex, and spinal reflexes without the forebrain and much of the brainstem. However, it is also clear that hypothalamic (and lower) level behavioral control is to varying degrees subject to cerebral cortical control, and that all behavior occurs in concert with the activity of the body as a whole (Mogenson et al., 1980; Swanson and Mogenson, 1981; Swanson, 2000; Canteras, 2018).

## Research Topic Contributions

Four of the included articles focus specifically on the spatially-extensive lateral hypothalamic area (LHA) that has received renewed attention in recent years, as successive inroads into its structural organization (Goto et al., 2005; Hahn, 2010; Hahn and Swanson, 2010, 2012, 2015; Canteras et al., 2011) have encouraged further forays into its functional roles (Leinninger, 2011; Li et al., 2011; Petrovich et al., 2012; Betley et al., 2013; Hsu et al., 2015). The first article, by Rangel et al., elucidates a novel role for an LHA region juxtaposed to the dorsomedial hypothalamic nucleus (the LHAjd), in relation to socially-relevant defensive behaviors; the second article, by Tyree and de Lecea, focuses on the relevance of LHA and ventral tegmental area (VTA) connections to the motor-output that is necessary for behavioral goal-seeking; the third article, by Petrovich, reviews recent evidence on the control of feeding behavior to support a view of the LHA as an interface between cognitive and sub-cognitive control; the fourth article, by Haller, delves into LHA involvement in aggression, and relates physiology to behavior, arguing the case that the LHA has a central role in deviant forms of aggressive behavior that are promoted by chronic glucocorticoid deficiency. In addition to these four LHA-related articles, a fifth, by Diniz and Bittencourt, relates broadly to them all as it provides a comprehensive and nicely illustrated review of the role of largely LHA-located melanin-concentrating hormone (MCH) neurons in relation to their participation in control of motivated behaviors.

Of the three remaining topic articles, one, by Hashikawa et al., also focuses on aggressive behavior: its neuroanatomical focus is the ventromedial hypothalamic nucleus (VMH), and a specific locus is the ventrolateral subdivision (VMHvl). Evidence to support a role for the VMHvl in generation of aggression is reviewed in relation to VMHvl neuronal connections. Hypothalamic connections are also the subject of an article by Micevych and Meisel, who focus their attention on circuit integration in relation to the control of female sexual behavior. Lastly, an article by Khan et al. demonstrates implementation of a novel computer-assisted method to facilitate interoperability between different brain atlases. To illustrate the approach (that has broad potential application), the authors use their hypothalamic datasets relating to behavioral control.

## Historical Perspectives

To round out this editorial are two illustrated perspectives (edited by JDH). The first, by George Fink, is broadly relevant to the topic, and the second, by Menno Kruk, relates more closely to some of the included articles. Both are historically-informed vignettes that serve to frame the included articles and the topic, and are also offered to inspire future research into hypothalamic structure and function.

### External Layer of the Median Eminence a Neurovascular Synapse

**Figure 2:**
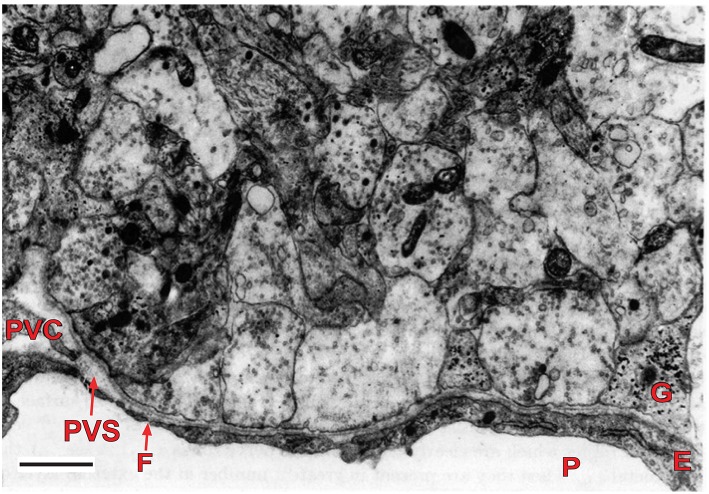
Electron micrograph of the external layer of the median eminence of a rat at the first postnatal day. Note the high density of nerve terminals, containing several agranular and granular vesicles, around part of a primary portal capillary vessel (P), which is fenestrated (F). The vesicles contain packaged neurohormone or neurotransmitter that undergo quantal release upon nerve depolarization resulting from action potentials. The neurohormones are released into the perivascular space (PVS), and from there they move rapidly into portal vessel blood for transport to the pituitary gland. This arrangement is typical of the neurohemal junctions found in the several circumventricular organs of the brain. Scale bar = 1 μm. E, endothelial cell; G, glial process; P, portal vessel; PVC, perivascular cell (reproduced with permission from Fink and Smith, 1971).

**Figure 3:**
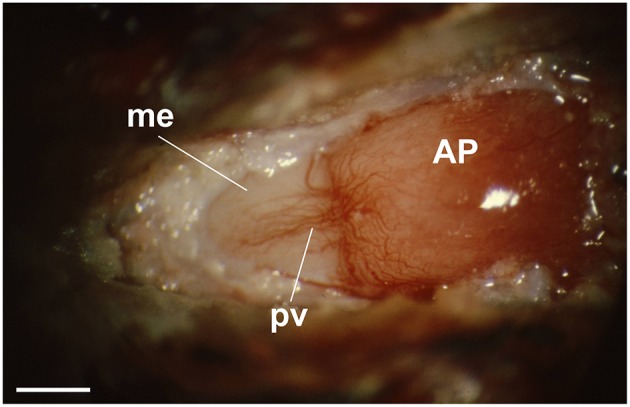
View through a dissecting microscope of the hypophysial portal vessels on the anterior surface of the pituitary stalk (left) of an anesthetized rat. The portal vessels (pv) (veins) arise from the primary capillary bed on the median eminence (me) (pink area to the left) and fan out over the anterior pituitary gland (AP) (right) at the me-AP junction. The tuberoinfundibular artery, a branch of the superior hypophysial artery, can be seen arching across the top of the me-AP junction, where it enters the AP. This artery passes through the anterior pituitary gland to supply arterial blood to the neurohypophysis. (reproduced with permission from Fink, 2012). Scale bar = ~500 μm.

The external layer of the median eminence (MEex) is comprised of hypothalamic neuron axons that terminate on the primary plexus of hypophysial portal vessels, where they form neurovascular synapses (Figure 2). This organization has been exploited experimentally as a model system for investigating central neurotransmission (Fink and Smith, 1971), and to investigate interactions between multiple different neurotransmitters expressed by different types of hypothalamic neurons whose axons converge in the MEex. This is exemplified by physiological and pharmacological studies on the release into hypophysial portal blood of several neurohormones, most of which are neuropeptides, such as gonadotropin-releasing hormone (GnRH) and corticotropin releasing factor (CRF) (Fink, 2012). However, non-peptide neurotransmitters such as dopamine, which inhibits prolactin release, are also released into hypophysial portal blood. The hypophysial portal vessels (Figure 3) convey these neurohormones to the anterior pituitary gland where they stimulate or inhibit the release of pituitary hormones (Fink, 2012).

It is possible to collect hypophysial portal vessel blood experimentally and thereby determine directly the characteristics of neurohormone/transmitter release under experimental conditions. The interaction of neurohormones is exemplified by the potentiation of CRF anterior pituitary signaling by arginine vasopressin (AVP) (Gillies et al., 1982; Sheward and Fink, 1991). Portal vessel blood measurements may also provide information on the processing of neuropeptide precursors and identify potentially novel signaling molecules (Antoni et al., 1992; Fink et al., 1992; Caraty et al., 2010; Clarke et al., 2012).

Direct measurements of GnRH in hypophysial portal blood confirmed the existence of the estrogen-induced ovulatory surge in spontaneously ovulating mammals (Sarkar et al., 1976; Sherwood et al., 1980; Caraty et al., 2010; Clarke et al., 2012), and demonstrated the way that estrogen feedback moderates pulsatile GnRH release (Sarkar and Fink, 1980; Clarke and Cummins, 1982; Fink, 2018). The latter explains why pulsatile gonadotropin release occurs in ovariectomized, but not intact, rhesus monkeys (Dierschke et al., 1970), and the differences in gonadotropin pulse frequency in post-menopausal compared with pre-menopausal women (Yen et al., 1972). Similarly, glucocorticoid negative feedback inhibition of adrenocorticotropic hormone (ACTH) secretion from the anterior pituitary gland, depending on its duration, is mediated by central moderation of CRF and AVP release as well as well as blockade of the pituitary response to CRF (Plotsky et al., 1986; Fink et al., 1988; Sheward and Fink, 1991).

The post-synaptic consequences of MEex neurovascular synaptic signaling can readily be determined by studying pituitary hormone release, which has elucidated novel mechanisms such as the self-priming effect of GnRH, by which the decapeptide can increase by several fold its effect on gonadotropin release, can enable small pulses of GnRH to induce an ovulatory gonadotropin surge, and has been used extensively in artificial insemination, animal husbandry, and fish farming (Fink, 1995, 2015).

### The Hypothalamic Ventromedial Nucleus: A Crucial Node in the Fight-Flight Balance?

Establishing that estrogen receptor-α (ESR1)-expressing neurons within the VMH ventrolateral part (VMHvl) are necessary and sufficient for aggressive behavior (Lin et al., 2011; Falkner et al., 2014; Kennedy et al., 2014) transformed the neuroscience of aggression, as it provided a specific locus from which to explore the “aggressive network” (Anderson, 2012; Yang et al., 2013, 2017; Hashikawa et al., 2016, 2017, 2018; Remedios et al., 2017; Hashikawa et al.). Other studies have identified cell groups in the amygdala and lateral septum that modify VMH activity (Choi et al., 2005; Wong et al., 2016). Moreover, activation of inhibitory (GABAergic) neurons in the medial amygdala can also elicit aggressive behavior (Hong et al., 2014). How the activity of these different cell groups is integrated is not fully understood, but a recent physiological experiment suggests a possible mechanism. Differential innervation of the “core” and “shell” of the VMH, directly from the basomedial amygdala, and indirectly from the anterior bed nucleus of the stria terminalis, produces “…a net inhibition or disinhibition of core neurons…depending on the firing rate of shell neurons,” imparting “…flexibility to this regulator of defensive and social behavior” (Yamamoto et al., 2018). Such flexibility might explain the episodic nature and context-sensitivity of fighting and underlie dynamic selection of appropriate behavioral responses in general (Brown et al., 1969b; Lammers et al., 1989; Haller et al., 1998a; Anderson, 2012; Yang et al., 2013, 2017; Hong et al., 2014; Kennedy et al., 2014; Hashikawa et al., 2016, 2018; Remedios et al., 2017; Todd et al., 2018; Todd and Machado, 2019).

**Figure 4:**
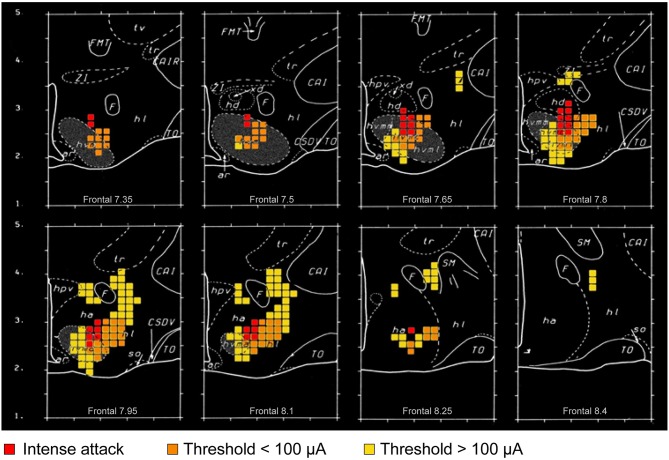
Graphic summary of mathematical analyses of the distribution of attack-eliciting electrodes in the rat hypothalamus (adapted and reproduced with permission from ref. Kruk et al., 1983). Colored square dimensions (as voxels) are 150 μm. Fiercest attacks (attack jumps) at the lowest current intensity are evoked from sites within and closely adjacent to the ventromedial hypothalamic nucleus (VMH) (red squares); whereas, milder attacks at low current intensity are evoked from a wider range of sites within and close to the VMH (orange squares). Yellow squares represent sites where attacks were elicited reliably at higher current intensities. The extensions (orange and yellow squares) of the “attack area” beyond the VMH somewhat overlap direct and indirect projections from parts of the amygdala, and cerebral cortex to the VMH and lateral hypothalamic area. It is noteworthy that the excitability of amygdala, hippocampal, and prefrontal cortical region neurons is subject to slow and rapid, as well as genomic and non-genomic, effects of corticosteroids (Joels et al., 2018). Impairing the adrenocortical stress response impairs elicited attacks, especially at sites indicated by the orange and yellow squares. Collectively, these findings suggest that corticosteroid effects on “fight-or-flight” responses in social conflict may be transmitted by amygdalar, hippocampal or prefrontal cerebral cortical connections to the VMH “core” and “shell.” Distances shown are mm (“Frontal” distances are relative to an interaural zero point). Abbreviations (for additional information see Kruk et al., 1983): ar, arcuate hypothalamic nucleus; CAI, internal capsule (R, rostral); CSOV, hypothalamic supraoptic decussations; F, fornix; FMT, mammillothalamic tract; ha, anterior hypothalamus (general region of); hd, dorsal hypothalamus (general region of); hl, lateral hypothalamus (general region of); hpv, hypothalamic paraventricular nucleus; hvmm, ventromedial hypothalamic nucleus, dorsomedial part; hvmc, ventromedial hypothalamic nucleus central part; hvml, ventromedial hypothalamic nucleus ventrolateral part; so, supraoptic nucleus; TO, optic tract; tr, reticular thalamic nucleus; tv, ventral thalamus; xd, dorsal region (of hd); ZI, zona incerta.

**Figure 5:**
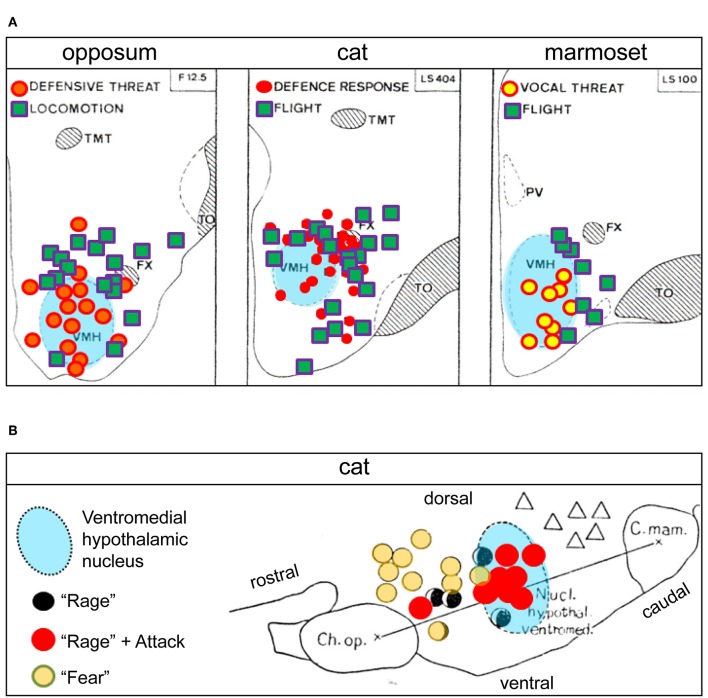
**(A)** Hypothalamic sites at which electrical stimulation elicited social conflict responses in three mammalian species: opossum, cat, and marmoset (adapted and reproduced with permission from Lipp and Hunsperger, 1978). The similar distribution of response sites for the three species suggests evolutionary conservation of “fight-or-flight” neuronal circuits at the level of the ventromedial hypothalamic nucleus (VMH). The different types of “social conflict” motor responses indicated reflect different aspects of “fight-or-flight” behaviors. Similar response site distribution patterns in the vicinity of the VMH have also been reported in rat and mouse (Lammers et al., 1988; Wong et al., 2016). **(B)** Comparative distribution from an earlier study of social conflict responsive sites (low-threshold) in the cat, shown in sagittal section (adapted and reproduced with permission from Yasukochi, 1960). Attacks and “rage” are elicited mostly within the VMH, while the response site for “rage” alone is shifted rostrally, and that for “fear” alone still further rostral in the hypothalamus, suggesting a VMH-centric circuit organization to control “fight-or-flight” behaviors in the cat (for additional perspective see Hinde, 1970). Ch. Op, optic chiasm; C. mam, mammillary body; FX, fornix; PV, paraventricular hypothalamic nucleus; TMT, thalamic mammillothalamic tract; TO, optic tract. Triangles in **(B)** = “yearning”.

Stimulation of the VMH and its surround is reported to evoke aggressive and defensive responses in several mammalian species (Yasukochi, 1960; Roberts et al., 1967; Brown et al., 1969a; Lipp and Hunsperger, 1978; Lammers et al., 1988; Kruk et al., 1983) (Figures 4, 5). However, predominant VHMvl association with overtly aggressive responses (Lin et al., 2011; Falkner et al., 2014, 2016; Kennedy et al., 2014) contrasts with VMH dorsolateral and central part association with defensive responses (Wang et al., 2015). This suggests the existence of a VMH-centric circuit for controlling opposing agonistic responses, echoing earlier ethological concepts of a mechanism for controlling “fight or flight” balance (Hinde, 1970).

In a manner similar to feedback (and feed-forward) control of the pituitary gland by circulating hormones (mentioned in the first perspective), the adrenocortical stress response (ACSR) (Joels et al., 2018) controls spontaneous and hypothalamus-elicited agonistic responses in experienced and inexperienced animals in different ways (Haller et al., 1998b, 2000a,b; Kruk et al., 1998, 2004, 2013; Mikics et al., 2007). An impaired ACSR tilts the balance toward “flight or freeze” in rats naïve to conflict but produces “pathological” attacks on opponents in bouts of spontaneous aggression (Haller et al., 2001, 2004). The behavioral changes correlate to altered hypothalamic excitability and enhanced amygdalar activity (Halasz et al., 2002; Kruk, 2014; Haller, 2018; Haller). A dynamic ACSR is clearly required for an adaptive response to social conflict. Interestingly, the absence of a well-timed ACSR in humans results in misguided aggression and poor conflict handling (Haller), possibly reflecting dysfunctional hypothalamic control.

## Concluding Remarks

The ability to perform motivated behaviors (purposeful movements) is a defining characteristic of animals. In this ability, with respect to the control of fundamental behaviors in mammals and other vertebrates, the hypothalamus takes center stage. The works of twentieth century ethologists, exemplified in those of Tinbergen (1951), paved a path that has led inexorably into the hypothalamus, and they continue to inspire neuroscientists interested in the study of behavior.

The current Research Topic, and the articles that comprise it, reflect ongoing and growing interest in the hypothalamus, driven partly by the increasing availability of investigative tools borne of molecular biology and computer science. However, with regard to those tools, Tinbergen's advocacy for observations of nature, rather than availability of technique, to direct one's research, seems prescient. More generally, current interest is also driven by a renewed recognition that a better understanding of hypothalamus structure and function has potential relevance for numerous diseases that impact the vital and varied physiological and behavioral functions in which the hypothalamus plays a central role (Hahn et al., 2019).

## Author Contributions

JH wrote the editorial. GF and MK provided illustrated perspectives (edited by JH). All authors reviewed the editorial and provided editorial guidance.

### Conflict of Interest Statement

The authors declare that the research was conducted in the absence of any commercial or financial relationships that could be construed as a potential conflict of interest.

## Footnotes

^1^The Oxford English Dictionary Dictionary (OED). “*motive, adj.”*. Oxford University Press.

^2^The Oxford English Dictionary Dictionary (OED). “*behave, v.”*. Oxford University Press.
